# Predictors of Mortality in COVID‐19 Patients Presenting With Gastrointestinal Bleeding: A Retrospective Cross‐Sectional Study

**DOI:** 10.1002/hsr2.72711

**Published:** 2026-06-28

**Authors:** Mobin Rasapour, Hamid Mohajer, Erfan Sabouri

**Affiliations:** ^1^ Clinical Research Development Center, Najafabad Branch Islamic Azad University Najafabad Iran; ^2^ Department of Internal Medicine, School of Medicine, Najafabad Branch Islamic Azad University Najafabad Iran

**Keywords:** COVID‐19, gastrointestinal bleeding, hospitalization, mortality

## Abstract

**Background and Aims:**

Coronavirus disease 2019 (COVID‐19), since its outbreak, has been a potentially fatal disorder. Gastrointestinal bleeding (GIB) with a notable prevalence of 1.5%–3% has been identified as a prominent cause of death in COVID‐19 patients. This study was conducted to evaluate GIB prevalence and mortality rate in COVID‐19 patients and to assess the role of potential risk factors on patient outcomes.

**Methods:**

In this retrospective cross‐sectional study, the records of RT‐PCR confirmed COVID‐19 patients who presented with overt GIB symptoms (melena, hematochezia, and hematemesis) in their course of admission or hospitalization in Isfahan Shariati Hospital, between March 2020 and 2022, were enrolled. After exclusion, data on patients' demographics, comorbid conditions, manifestations of GIB, bleeding onset, and anticoagulant administration were extracted. Subjects were divided into two groups: alive and deceased 30 days after discharge to assess the role of variables on mortality.

**Results:**

Among 7243 COVID‐19 patients, 107 (1.5%) individuals experienced GIB symptoms. After excluding patients with GIB not attributable to COVID‐19, 63 patients (0.86%) were included in the analysis, of whom 47.6% were reported deceased. The most common manifestations were hematemesis (42.9%) and melena (33.3%), indicating 76.2% of the upper GIB source. 61.9% of GIBs occurred during hospitalization, and the prevalence of hypertension, diabetes, and cardiovascular disease was 39.7%, 31.7%, and 19%, respectively. Endoscopic evaluation was performed in 12 patients, with gastric and duodenal ulcers being the most frequent findings (5 cases, 41.6%). Other than a significant association between the outcomes and the onset of bleeding (*p* = 0.001, OR = 5.53), no statistically significant relationship was found among other variables.

**Conclusion:**

GIB developing during hospitalization, as opposed to bleeding that occurred prior to admission, was suggested as a prognostic factor of mortality in COVID‐19 patients. Considering the fatal consequences of GIB in COVID‐19 patients, further studies are recommended to assess the roles of risk factors in preventing adverse outcomes.

## 1 Introduction

Since its outbreak, Coronavirus disease 2019 (COVID‐19) has afflicted more than 700 million patients and led to over 7 million deaths attributed to its complications [1, 2]. These substantial mortality rates are primarily driven by severe acute respiratory distress syndrome, cardiac arrhythmia, cardiac arrest, and pulmonary embolism [3]. To a lesser extent, gastrointestinal bleeding (GIB), with an estimated prevalence of 1.5% to 3% in COVID‐19 patients, has also been reported as a contributing factor to patient fatality [4, 5]. Several potential mechanisms have been proposed to elucidate the pathological process leading to the occurrence of GIB and its adverse consequences on COVID‐19 patients [5, 6, 7, 8]. The severe acute respiratory syndrome coronavirus 2 (SARS‐CoV‐2) is not only capable of directly invading enterocytes by binding to angiotensin‐converting enzyme 2 (ACE2) receptors, but also triggers stress‐related mucosal disease because of the severe systemic inflammatory response triggered by immune cells [9, 10]. In addition, the pro‐coagulant state associated with COVID‐19 increases the risk of thrombosis, which leads to ischemic injury and mucosal ulceration throughout the gastrointestinal tract [10]. Furthermore, medications such as corticosteroids, anticoagulants, and antiplatelet agents, which are frequently administered to COVID‐19 patients, increase the risk of GIB by decreasing the synthesis of protective prostaglandins, reducing endogenous epithelial repair mechanisms, and inhibiting clotting factors [11, 12, 13, 14, 15, 16]. These mucosal injuries may lead to an acute, fatal state of severe bleeding in the gastrointestinal tract, which alters the hemodynamics of patients and induces hypovolemic shock [17]. In patients with milder states of mucosal injury, the occult loss of blood leads to a decrease in hemoglobin concentration, which in turn reduces oxygen transport, resulting in tissue hypoxia and multiple organ dysfunction [18, 19].

Some previous studies on non‐COVID‐19 patients with GIB have identified age, gender, diabetes mellitus (DM), cardiovascular diseases (CVD), and upper sources of GIB as significant risk factors for higher mortality rates [20, 21, 22, 23, 24]. Moreover, age, gender, DM, and CVD are also indicated as risk factors for fatal outcomes in COVID‐19 patients generally [24, 25, 26]. Due to a lack of literature to determine the risk factors for mortality in COVID‐19 patients who experienced GIB as a complication of their disease, this study was conducted to evaluate the mortality rates of COVID‐19 patients who manifested GIB in their disease course and to identify potential risk factors for mortality in these patients.

## 2 Materials and Methods

### 2.1 Study Design

This study was conducted as a retrospective cross‐sectional analysis on the records of all COVID‐19 patients over 18 years of age who were admitted to Isfahan Shariati Hospital between March 2020 and March 2022. Considering the ethical guidelines, approved by the Najafabad University Ethics Committee (IR.IAU.NAJAFABAD.REC.1401.146), informed written consent had previously been obtained from each individual, allowing access to their medical records.

### 2.2 Data Collection

COVID‐19 patients were selected based on the condition of having a positive reverse transcriptase–polymerase chain reaction (RT‐PCR) test on nasal or throat samples taken at least 1 week prior to or during their hospital admission. All the records of confirmed COVID‐19 patients were carefully evaluated for any presentations of overt GIB (e.g., hematemesis, melena, and hematochezia) in the course of their disease, both prior to and during their hospital stay. To avoid bias in data collection and minimize the chance of missing records with GIB manifestations, two trained medical doctors independently assessed each record. The records of patients with any prior history of GIB, peptic ulcer disease, diverticulitis, hepatic cirrhosis, inflammatory bowel disease, end‐stage renal disease, or gastrointestinal malignancies were excluded from the study.

Data on age, gender, the presence of comorbid conditions (e.g., hypertension, DM, and cardiovascular disease), the initial manifestation of GIB (e.g., hematemesis, melena, and hematochezia), the onset of GIB symptoms (before or during hospitalization), endoscopic findings (if performed), and the administration of anticoagulants during hospitalization (as prophylaxis for hypercoagulable state) were extracted from the medical and drug histories documented on admission and progress notes. Patients were considered hypertensive or diabetic if they were on related medical prescriptions prior to hospitalization, and considered to have cardiovascular disease if they had reported a history of myocardial infarction, stroke, heart failure, or cardiomyopathies. Anticoagulant usage was assumed when patients received unfractionated heparin, low molecular weight heparins, vitamin K antagonists, or direct thrombin and factor Xa inhibitors during their hospital stay. Another aspect of patients investigated in this study was the source of their bleeding, which was established based on their manifestation of GIB, with melena or hematemesis indicating an upper GIB (bleeding from the esophagus up to the Treitz ligament) and hematochezia indicating a lower GIB (bleeding distal to the Treitz ligament) [27]. Additionally, patient outcomes, specifically whether they were alive or deceased 30 days following discharge, were determined through phone interviews with patients themselves or their family members.

### 2.3 Statistical Analysis

All the mentioned data were analyzed using IBM SPSS Statistics (Version 26). The Kolmogorov‐Smirnov test was used to determine the normality of the data distribution. Patients were divided into two groups based on their outcomes (alive or deceased), and the role of each variable was assessed between the groups. Statistical inference was based on chi‐square or Fisher's exact test for categorical variables and independent *t*‐test for continuous variables. Variables that were statistically significant in univariate analysis (*p* < 0.05) were included in a binary logistic regression model to identify independent predictors. All statistical tests were two‐sided, and a *p* < 0.05 was considered statistically significant.

## 3 Results

**Figure 1:**
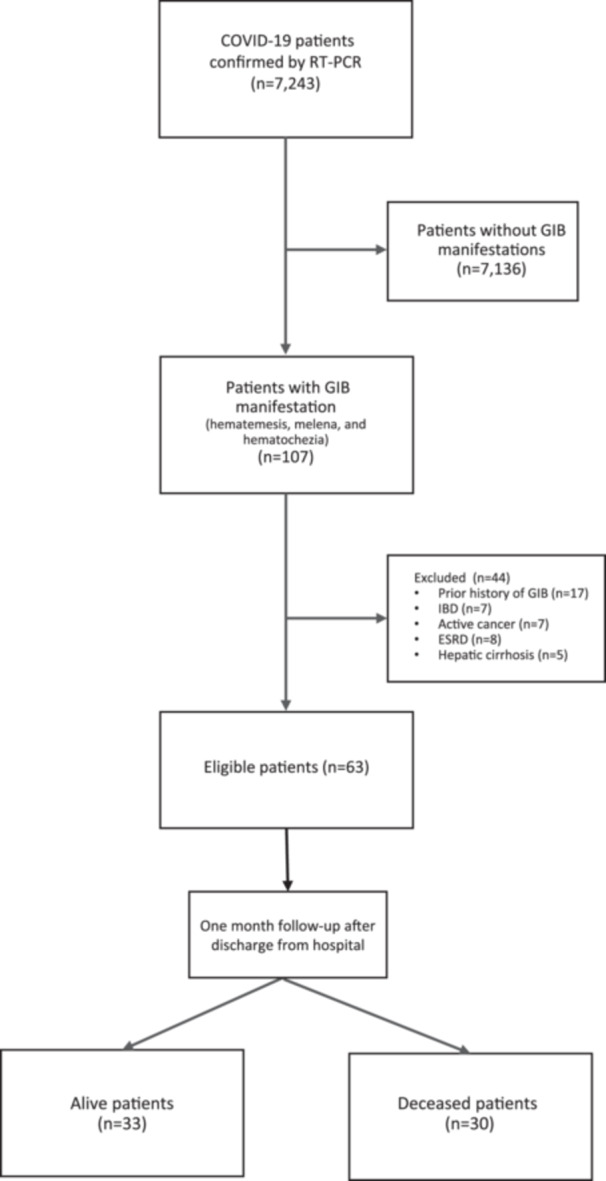
Diagram of the study enrollment and patient outcomes. COVID‐19, Coronavirus disease 2019; ESRD, End‐stage renal disease; GIB, Gastrointestinal bleeding; IBD, Inflammatory bowel disease; RT‐PCR, Positive reverse transcriptase–polymerase chain reaction.

In this study, 7243 patients were diagnosed with COVID‐19, of whom 107 (1.5%) experienced GIB during the course of their illness. After excluding patients with previously mentioned diseases, 63 individuals (0.86%) remained in the study whose GIB symptoms could not be attributed to any etiology other than COVID‐19 (Figure 1).

Among the 63 patients ultimately included in the study, the mean age was 66.66 ± 13.51 years, within a range of 28–91 years, of which 71.4% were male. In 61.9% of the patients, the onset of GIB symptoms occurred during hospitalization, with the most common manifestations being hematemesis (42.9%), and melena (33.3%), indicating that 76.2% of patients had an upper source of GIB. The prevalence of hypertension, DM, and cardiovascular disease in the study population was 39.7%, 31.7%, and 19%, respectively. Additionally, 57.1% of the patients reported having at least one of these comorbid conditions. Furthermore, within this patient group, 81.0% received anticoagulant therapy as a preventive measure against thrombotic complications. Following the subsequent evaluation, the mortality rate among COVID‐19 patients who experienced GIB during their disease course was 47.6%.

No statistically significant relationship was found between the outcome of patients and their age, source of the GIB (categorized as upper and lower), initial manifestations of GIB, administration of anticoagulants during hospitalization, and comorbid conditions (e.g., hypertension, DM, and cardiovascular disease). However, there was a significant association between female gender and mortality (*p* = 0.01), and a highly statistically significant association between mortality and the onset of bleeding during hospitalization (*p* = 0.001) (Table 1). Furthermore, endoscopic evaluation was performed in 12 patients (19%); this included eight upper (66.6%) and four lower (33.3%) endoscopies. The most common findings were gastric or duodenal ulcers (41.6%), as presented in Table 1. Endoscopic findings were not significantly associated with mortality.

**Table 1:** Comparison between the potential risk factors and the outcome of patients.

Risk factors		Total	Outcome		*p*‐value
			Alive	Dead	
Age (mean ± SD)	—	66.66 ± 13.51	65.21 ± 14.05	68.27 ± 12.94	0.37^a^
Gender, *n* (%)	Female	18 (28.6)	5 (15.2)	13 (43.3)	**0.01** b
	Male	45 (71.4)	28 (84.8)	17 (56.7)	
Comorbid conditions, *n* (%)	Overall presence	36 (57.1)	17 (51.5)	19 (63.3)	0.34^b^
	CVD	12 (19)	5 (15.2)	7 (23.3)	0.41^b^
	DM	20 (31.7)	11 (33.3)	9 (30)	0.78^b^
	HTN	25 (39.7)	12 (36.4)	13 (43.3)	0.57^b^
Manifestation, *n* (%)	Melena	21 (33.3)	12 (36.4)	9 (30)	0.59^b^
	Hematemesis	27 (42.9)	13 (39.4)	14 (46.7)	0.56^b^
	Hematochezia	15 (23.8)	8 (24.2)	7 (23.3)	0.93^b^ 0.82^b^
Source, *n* (%)	LGIB	15 (23.8)	8 (24.2)	7 (23.3)	0.93^b^
	UGIB	48 (76.2)	25 (75.8)	23 (76.7)	
Bleeding onset, *n* (%)	Before hospitalization	24 (38.1)	19 (57.6)	5 (16.7)	**0.001** ^b^
	During hospitalization	39 (61.9)	14 (42.4)	25 (83.3)	
Anticoagulant administration^c^, *n* (%)	Yes No	38 (97.4) 1 (2.6)	13 (92.9) 1 (7.1)	25 (100) 0 (0)	0.36^b^
Endoscopic findings, *n* (%)	Overall reports Normal Gastric or duodenal ulcer Gastritis/erosion Internal hemorrhoid	12 (100) 2 (16.6) 5 (41.6) 3 (25) 2 (16.6)	9 (100) 1 (11.1) 4 (44.4) 2 (22.2) 2 (22.2)	3 (100) 1 (33.3) 1 (33.3) 1 (33.3) 0 (0)	1.00^d^

*Note:* Bold values indicate statistical significance at *p* < 0.05.

Abbreviations: CVD, cardiovascular disease; DM, diabetes mellitus; HTN, hypertension; LGIB, lower gastrointestinal bleeding; SD, standard deviation; UGIB, upper gastrointestinal bleeding.

^a^ Independent *t*‐test.

^b^ Chi‐square test.

^c^ The comparison was limited to patients who developed GIB during their hospital stay, since prior onset of GIB would not be due to the administered anticoagulant medications.

^d^ Fisher's exact test (small sample size limits interpretation).

Further analysis using logistic regression models was performed to investigate the effects of potential predictors on mortality (Table 2), ensuring that the association of each variable was assessed independently of the others. In this evaluation, although higher mortality rates were observed among female patients, the increase was only statistically significant in those whose onset of bleeding occurred during their hospitalization. (*p* = 0.006, OR = 5.53, 95% CI [1.641, 18.645]).

**Table 2:** Logistic regression models for the effect of potential predictors on mortality.

	B	S.E.	Sig	OR	C.I. 95% for OR	
					Lower	Upper
Gender						
Female	REF	REF	REF	REF	REF	REF
Male	−1.104	0.652	0.09	0.331	0.092	1.189
Bleeding onset						
Before hospitalization	REF	REF	REF	REF	REF	REF
During hospitalization	1.711	0.620	**0.006**	5.53	1.641	18.645

*Note:* Bold values indicate statistical significance at *p* < 0.05.

Abbreviations: B, logistic regression coefficient (log odds); C.I., confidence interval; GIB, gastrointestinal bleeding; OR, odds ratio; S.E., standard error.

## 4 Discussion

In this study, out of 7243 confirmed COVID‐19 patients, 107 individuals (1.5%) experienced GIB symptoms during their disease course. Among these, only 63 patients (0.9%) matched our inclusion criteria of not having any prior disease that could be responsible for the GIB presentation. With a high mortality rate of 47% among these patients (30/63), it was established that gender and the onset of bleeding during hospitalization were the factors most notably associated with increased risk of mortality. However, after adjusting for confounding variables, only the onset of bleeding remained significantly associated. Based on our findings, age, source, and manifestation of bleeding, administration of anticoagulants, and presence of underlying conditions (e.g., diabetes, hypertension, and cardiovascular disease) could not be considered as predictors of mortality in COVID‐19 patients with GIB.

Previous studies have reported a wide range of 0.47%–19% GIB prevalence in COVID‐19 patients, which is justifiable by the characteristics of the studied populations and the GIB screening methods used [28]. A higher prevalence of GIB is observed in patients who receive anticoagulants and in those with more severe COVID‐19, typically admitted to the ICU [29, 30, 31]. This is further evidenced by a meta‐analysis of 33 studies encompassing 134,905 COVID‐19 patients, in which it was found that the pooled prevalence of GIB was 3.05%, rising to 6.2% when only anticoagulant patients were included [28]. Furthermore, GIB categorization of COVID‐19 patients solely based on gastrointestinal manifestations generally shows a prevalence of 1.5%–3% [4]. On the other hand, using diagnostic laboratory tests such as occult blood tests on fecal or gastric samples reveals a more notable prevalence of 11.7% in these patients [32]. Our study's 1.5% GIB prevalence aligns with the mentioned previous findings and the largest study to date, in which a 1.87% rate of GIB was reported among 1,050,045 COVID‐19 patients [33].

Mortality rates in COVID‐19 patients experiencing GIB have previously been reported in numerous studies, mainly suggesting a wide range of 20%–39.5% [33, 34, 35, 36, 37, 38]. While in a study on 76 patients, in‐hospital mortality was reported to be 20% [38], in the study by Aldiabat et al. on 19,715 COVID‐19 patients hospitalized in the United States with nonvariceal upper GIB, the mortality rate was reported as 33.7% [33]. The mortality rate reported in our study (47.6%) was relatively higher than in the mentioned studies; however, in a study on 43 occult blood positive patients, an even higher rate (74.4%) was reported [32]. These variations in mortality rates can be attributed to differences in the selected population, ethnicity, the duration of patients' follow‐up, the quality of care, and the most common virus strains during the studies.

Although this is the first study to our knowledge that aimed to evaluate gender as a prognostic risk factor for mortality in COVID‐19 patients experiencing GIB, the association between gender and mortality in COVID‐19 patients or GIB patients individually has already been discussed in previous studies. In COVID‐19, patients of male gender are reported to experience significantly higher fatality rates across all age groups [39, 40]. This is mostly attributable to higher prevalence of underlying conditions such as cardiovascular disease, weaker immune responses, and higher rates of smoking and alcohol consumption in males [41]. On the other hand, although the effects of gender on the mortality rates of patients with upper GIB remain controversial [20, 24, 25, 42], it is suggested that male patients with lower GIB tend to have significantly higher mortality rates than females (4.5% vs. 3.4%) [23]. In our study, although female gender was surprisingly found to be associated with higher mortality rates using non‐parametric statistical tests, further analysis with logistic regression models revealed this association to be insignificant. This suggests that other variables, such as age, bleeding onset, and comorbid conditions, could be considered as confounders that have to be accounted for in future analyses.

Age, particularly those over 50, is one of the most prognostic risk factors for mortality in COVID‐19 patients [43]. This is attributed to several factors, including a higher prevalence of comorbidities, diminished reserve capacity of vital organs, and a decreased immune system capacity in aged individuals [44]. Furthermore, in non‐COVID patients with lower GIB, aged patients were reported to have significantly higher mortality rates, whereas in patients with upper GIB, the effect of age on mortality remains controversial [23, 24, 25, 42]. In this study, which to our knowledge is also the first to evaluate age as a prognostic risk factor for mortality in COVID‐19 patients experiencing GIB, although the average age of the deceased group was higher than that of the surviving subjects, this difference was found to be not statistically significant.

In patients with COVID‐19, underlying conditions such as hypertension, DM, and cardiovascular disease have been identified as risk factors for more severe disease and fatal outcomes [26]. On the other hand, although studies suggest that non‐gastrointestinal comorbidities are independent risk factors for GIB, controversy remains regarding whether these comorbidities are associated with mortality [23, 45]. For example, a systematic review of 808 COVID‐19 patients found that hypertension was associated with increased mortality among those with GIB [46]. In contrast, a study of 170 patients with upper GIB found that none of the three aforementioned underlying conditions were significant predictors of 60‐day mortality [24]. Our findings support the latter study, showing no significant correlation between these conditions and mortality.

Assessing the source of GIB in COVID‐19 patients has previously demonstrated that upper GIB is more prevalent than lower GIB in COVID‐19 patients [28]. Moreover, in non‐COVID‐19 patients, higher mortality rates from upper GIBs have been reported than in patients with lower GIBs [21, 22, 23]. Consistent with these findings, patients with upper GIB exhibited a slightly higher mortality rate than those with lower GIB in our study (47.9% vs. 46.6%), although the difference was not statistically significant.

The onset of GIB has previously been linked to patient outcomes, as patients developing GIB during their hospital stay have significantly higher mortality rates than those who develop GIB prior to their admission [47, 48]. Particularly, a multicenter study assessing risk factors in 314 COVID‐19 patients experiencing GIB demonstrated that for patients who developed GIB during hospitalization, there was an increased mortality risk (OR = 1.58, *p* = 0.02) [49]. In corroboration with these results, in our study, both non‐parametric statistical tests and a binary logistic regression model revealed that the mortality rate in patients who developed GIB during hospitalization is significantly higher than in those with GIB manifestations prior to admission. This increased mortality rate in hospitalized patients may be attributed to the severity of the disease, stress‐related mucosal ulcers, or the impact of medical treatments. However, there is a necessity for further detailed studies to identify the causes of these observations.

Thromboprophylaxis with anticoagulants is recommended in all hospitalized adults with COVID‐19, since the disease is known to be associated with a hypercoagulable state due to endothelial damage of vessels [50]. However, it is widely recognized that anticoagulant therapy increases the risks of bleeding and trends towards higher inpatient death, therefore requires close clinical monitoring [11, 12]. To assess the role of anticoagulant therapy on the risk of adverse outcomes, a previous study on 146 hospitalized COVID‐19 patients with GIB during their admission surprisingly found that initiating anticoagulant therapy protocols has a protective effect against mortality [36]. Moreover, in a propensity score‐matched case‐control study on 314 COVID‐19 patients with GIB, use of anticoagulation medication or antiplatelet agents was not associated with increased risk of GIB in COVID‐19 patients [49]. Consistent with these findings, in this study, anticoagulant prophylaxis had no significant effect on the mortality of COVID‐19 patients who developed GIB during their hospitalization.

Due to the extraordinary circumstances imposed by the COVID‐19 pandemic, the frequency of endoscopic evaluations was reduced [51, 52]. In a systematic review and meta‐analysis of 1458 patients with GIB, only 286 individuals (19.6%) underwent endoscopy, with gastric or duodenal ulcers being the most common findings (117 cases, 40.9%) [28]. Likewise, in another systematic review of 808 COVID‐19 patients, ulcers were the most common finding along the gastrointestinal tract [46]. A similar reduction in endoscopic procedures was observed in our cohort, where 12 patients (19%) underwent endoscopic evaluation, and gastric or duodenal ulcers were the most frequent findings (5 cases, 41.6%). With respect to lower gastrointestinal findings, internal hemorrhoids were the only abnormality identified in our cohort. However, given the aforementioned reduction in endoscopic procedures, these results should be interpreted with caution. By comparison, among 20 colonoscopies performed on 38 COVID‐19 patients, segmental colitis associated with diverticulitis was the most prevalent finding, potentially pointing to an underlying inflammatory process [53]. None of the endoscopic findings were significantly associated with mortality. However, it should be noted that the number of patients who underwent endoscopy in our study was limited; therefore, these results should be interpreted with caution.

Although our study, with its originality, considerable population size, notable amounts of follow‐up days, sufficient duration of the study for inclusion of all SARS‐CoV‐2 strains pandemics, variety of assessed risk factors, and the strict overview of patient records, indicates reliable insightful findings, there are a few limitations that have to be taken into consideration as well. The retrospective nature of the study, the lack of ethnic diversity in the study population, possible inaccuracy of self‐reported information, possible categorization error of variables despite the recruitment of two trained medical doctors, the inability to apply laboratory tests, and the reduced rate of endoscopic examinations could be considered as limitations of our study.

## 5 Conclusions

In conclusion, GIB with its high prevalence and associated mortality rate represents a critical condition in COVID‐19 patients that requires more extensive study. Our findings suggest that the onset of bleeding during hospitalization can act as a prognostic factor in COVID‐19 patients with GIB. However, further prospective studies are needed to validate our findings and explore whether or not performing endoscopy reduces the risk of death, considering the challenges of performing it in COVID‐19 patients. This insight will aid clinicians in the management of COVID‐19 and future infectious diseases that might result in comparable severe pandemics.

## Author Contributions

**Mobin Rasapour:** data curation, writing – original draft, investigation. **Hamid Mohajer:** conceptualization, supervision. **Erfan Sabouri:** writing – review and editing, formal analysis, project administration.

## Funding

The authors have nothing to report.

## Conflicts of Interest

The authors declare no conflicts of interest.

## Transparency Statement

Erfan Sabouri affirms that this manuscript is an honest, accurate, and transparent account of the study being reported; that no important aspects of the study have been omitted; and that any discrepancies from the study as planned (and, if relevant, registered) have been explained.

## Data Availability

The data that support the findings of this study are available from the corresponding author upon reasonable request.
